# Dynamics of sterol synthesis during development of *Leishmania* spp. parasites to their virulent form

**DOI:** 10.1186/s13071-016-1470-0

**Published:** 2016-04-12

**Authors:** Chaoqun Yao, Mary E. Wilson

**Affiliations:** Department of Biomedical Sciences and One Health Center for Zoonoses and Tropical Veterinary Medicine, Ross University School of Veterinary Medicine, P.O. Box 334, Basseterre, St. Kitts, West Indies ᅟ; Departments of Internal Medicine, Microbiology and Epidemiology, University of Iowa, Iowa City, IA USA; Iowa City VA Medical Center, Iowa City, IA USA

**Keywords:** *Leishmania*, Sterol, Promastigotes, Metacyclogenesis, Virulence, Sterol biosynthetic pathway

## Abstract

**Background:**

The *Leishmania* spp. protozoa, the causative agents of the “neglected” tropical disease leishmaniasis, are transmitted to mammals by sand fly vectors. Within the sand fly, parasites transform from amastigotes to procyclic promastigotes, followed by development of virulent (metacyclic) promastigote forms. The latter are infectious to mammalian hosts. Biochemical components localized in the parasite plasma membrane such as proteins and sterols play a pivotal role in *Leishmania* pathogenesis. *Leishmania* spp. lack the enzymes for cholesterol synthesis, and the dynamics of sterol acquisition and biosynthesis in parasite developmental stages are not understood. We hypothesized that dynamic changes in sterol composition during metacyclogenesis contribute to the virulence of metacyclic promastigotes.

**Methods:**

Sterols were extracted from logarithmic phase or metacyclic promastigotes grown in liquid culture with or without cholesterol, and analyzed qualitatively and quantitatively by gas chromatograph-mass spectrometry (GC-MS). TriTrypDB was searched for identification of genes involved in *Leishmania* sterol biosynthetic pathways.

**Results:**

In total nine sterols were identified. There were dynamic changes in sterols during promastigote metacyclogenesis. Cholesterol in the culture medium affected sterol composition in different parasite stages. There were qualitative and relative quantitative differences between the sterol content of virulent versus avirulent parasite strains. A tentative sterol biosynthetic pathway in *Leishmania* spp. promastigotes was identified.

**Conclusions:**

Significant differences in sterol composition were observed between promastigote stages, and between parasites exposed to different extracellular cholesterol in the environment. These data lay the foundation for further investigating the role of sterols in the pathogenesis of *Leishmania* spp. infections.

**Electronic supplementary material:**

The online version of this article (doi:10.1186/s13071-016-1470-0) contains supplementary material, which is available to authorized users.

## Background

*Leishmania* spp. are the etiological agents of leishmaniasis, a group of parasitic diseases that are endemic in 88 countries on four continents [1]. Over 20 *Leishmania* spp. are collectively responsible for varied clinic manifestations of human disease. Three major clinical forms include cutaneous leishmaniasis (CL), mucocutanous leishmaniasis and visceral leishmaniasis (VL). Data compiled by WHO show one million cases of CL in the last five years, and 300,000 cases of VL with 20,000 deaths annually (http://www.who.int/leishmaniasis/en/).

The *Leishmania* spp. protozoa alternate between a flagellated promastigote in the sand fly vector and an obligate intracellular amastigote, which lacks an external flagellum, in the mammalian host. After a sand fly vector takes a blood meal, amastigotes transform in the sand fly gut to procyclic promastigotes and several other intermediate stages, multiply by binary fission, and eventually develop to the metacyclic promastigotes which are infectious for mammalian hosts. Metacyclic promastigotes are inoculated by the sand fly vector during a blood meal into a new mammalian host [2]. This process of development from the procyclic to the metacyclic promastigotes is termed metacyclogenesis. Due to the technical challenges in raising sand flies in laboratory, metacyclogenesis is often modeled in liquid medium by culture of promastigotes in vitro from logarithmic to stationary growth phase, from which the metacyclic promastigotes can be isolated by density for some *Leishmania* spp. to more than 95 % purity [3]. Metacyclic promastigotes are morphologically distinguishable from procyclic cells by their elongated flagellum (at least double the cell body length), and their smaller body size.

Unlike mammalian cells but similar to fungi, *Leishmania* spp. are eukaryotic kinetoplastids that synthesize ergosterol [4]. They do not have the enzymes to synthesize cholesterol, although they have detectable cholesterol that they must take up from their external environment [5]. The sterols ergosterol or cholesterol are essential components of plasma membranes found in lipid rafts, membrane microdomains which are also classified as detergent resistant membranes (DRMs) due to their physicochemical properties [6, 7]. Our prior studies using the lipid-raft disrupting agent methyl-β-cyclodextrin (MβCD) suggested that lipid rafts play a pivotal role in the virulence of *Leishmania* spp. [8]. The anti-fungal agent amphotericin B is widely used in patients with leishmaniasis, especially in regions where parasites are resistant to standard therapy with antimony compounds [9, 10], or in patients co-infected with *Leishmania* sp. and human immunodeficiency virus (HIV) [11]. Amphotericin B preferentially binds to ergosterol, leading to disruption of the osmotic integrity of the membrane in target cells [12].

Given the association between sterol content and heat resistance of *L. major* [13], the objectives of current study were (i) to identify and quantify sterols in distinct stages of *Leishmania infantum* promastigotes that differ in their virulence for a mammalian host, and (ii) to determine the effects of cholesterol addition or depletion on promastigote sterol (particularly ergosterol) content. The data should provide a baseline for further study of drug- or environmentally-induced changes in parasite sterol content in the pathogenesis and control of *Leishmania* spp. infections.

## Methods

### Ethics Statement

The Animal Care and Use Committee of the Iowa City Veterans’ Affairs Medical Center reviewed and approved protocols for procedures used (Protocols number 1190301 and 1190302). The animal care and use were in accordance with the recommendations in the Guide for the Care and Use of Laboratory Animals of the National Institute of Health.

### Parasites

A Brazilian strain of *Leishmania infantum* (previously called *L. chagasi*) (MHOM/BR/00/1669) was serially passaged in golden hamsters to maintain parasite virulence. Amastigotes were isolated from infected hamster spleens and allowed to transform to promastigotes in HOMEM (Hemoflagellate modified Minimal Essential Medium) with 10 % fetal calf serum (FCS) and hemin made from commercially available ingredients as previously described [8]. Serum-free medium (SFM) was prepared similar to HOMEM but lacking FCS and with additional biopterin, adenosine and bovine serum albumin. Virulent promastigotes were used within three passages post-isolation from hamster. An avirulent strain L5 was derived from the same *L. infantum* isolate by serial passage in vitro for over five years as described [14].

Cultures of virulent or avirulent promastigotes were seeded at 1 × 10^6^ cells/ml in HOMEM or SFM, and allowed to grow at 26 °C to the appropriate cell density, monitored daily by microscopic exam on a hemacytometer. Metacyclic promastigotes were isolated from Day 8 stationary-phase cultures of virulent strain parasites on a discontinuous Ficoll gradient as described [3]. Metacyclic promastigotes could not be recovered from similar cultures of avirulent promastigotes (data not shown). In some experiments, metacyclic promastigotes were incubated in 25 mM MβCD (Sigma, St. Louis, MO) freshly prepared in RPMI 1640 (GIBCO) at a cell density of 2 × 10^8^ cells/ml at room temperature for 1 h to deplete cellular sterols, and then washed twice by centrifugation in Hanks’ balanced salt solution (GIBCO) as described [8, 15].

### Sterol isolation from promastigotes

Sterols were extracted from logarithmic phase, stationary phase or metacyclic promastigotes with dicholoromethane and methanol (2:1 ratio [vol/vol]; Sigma) by sequential centrifugation according to a published protocol [16] with some modifications as previously described [8]. Dry sterol aliquots from 2 × 10^8^ promastigote equivalents were maintained at -20 °C immediately after extraction until a single use. Extracted sterols were saponified in 30 % KOH in methanol and derivatized with dichloromethane and bis (trimethylsilyl) trifluroacetamide (BSTFA) (Sigma) in 1:2 ratio following a previously-described protocol [8].

### Sterol identification

Dry derivatized trimethylsilyl derivatives were analyzed in the High Resolution Mass Spectrometry Facility at the University of Iowa using a ThermoFinnigan Voyager single quadruple mass spectrometer interfaced with a Trace2000 gas chromatograph (GC). The original software from the supplier was used for both data acquisition and processing. A DB-5ht capillary column (inner diameter, 0.25 mm; length 30 m) (JW Scientific Inc., Folsom, CA) was used. The GC temperature program settings were 175 °C for 1 min, ramp up at 10 °C/min to 280 °C, and hold at 280 °C for 30 min. The GC inlet temperature was set at 280 °C. The GC eluent was electronically ionized at 70 eV. The mass spectrometer was programmed to detect a mass range of 45–700 Da.

Sterol identification was achieved by two independent methods. First, the GC retention time of same chemical compound is very consistent under the same experimental conditions (GC column, solvent, temperature etc.). Therefore, known chromatograph grade cholesterol and ergosterol standards (Sigma) were used every time to generate spectra, used to identify the corresponding sterol in individual samples by comparison. For ergosterol-related compounds lacking appropriate standards, the GC retention time was used once identified (see below). Second, the unique mass spectrum of each GC peak was searched against the small-molecule library (2001 version) of the National Institute of Standards and Technology library (NIST)/EPA/NIH Mass Spectral Library, which was purchased with the GC-MS instrument. The library search program was integrated in the Xcalibur operating software. Positive identification of a sterol was made if its mass spectrum matched a sterol in the library with a SI/RSI matching factor score ≥780 at a probability score of at least 50.

### Sterol quantification

Two approaches were used to quantify sterols, capable of either relative or absolute quantification. Multiple preliminary trials showed that either cholesterol or ergosterol amounts were proportionally and linearly correlated with the area under its respective sterol peak on GC spectra. Thus the area under peaks was used for relative quantification of sterol components. For each promastigote stage, the total amount of all sterols added up to 100 %.

The absolute amount of cholesterol was also quantified as follows. A standard curve was generated for each experiment using varying amounts of chromatograph-grade cholesterol that was deuterated at six positions ([^2^H]cholesterol; Sigma), and varying amounts of chromatograph-grade ergosterol (Sigma). Quality control for each experiment was achieved by comparing multiple standard curves run at the time of each experiment, requiring a correlation factor of 0.98 or higher between standard amounts and retention times/area under curves. Samples and standards that did not meet quality control were rerun. Known amounts of [^2^H] cholesterol were also spiked into each sample, and samples were analyzed at three levels of cell equivalents to make sure [^2^H] cholesterol from at least two cell equivalents were within the range of the standard curve. The absolute amount of unknown cholesterol in each sample was determined by its area relative to that of [^2^H] cholesterol standards. Amounts of ergosterol and other ergosterol-related sterols for which deuterated standards were not available were calculated considering their relative abundance compared to the absolute amount of cholesterol in the same sample. The peak areas under curves were used to generate the extrapolated values.

### Sterol biosynthetic pathway (SBP) of *Leishmania* spp.

SBP genes of *Trypanosoma cruzi* have been identified in a recent publication [17]. Locus identifiers of *T. cruzi* SBP genes were used as gene ID to search TriTryp database (http://tritrypdb.org/tritrypdb/, latest access date 01/30/16), resulting in identification *L. major* homologues, which were used to pull out all *Leishmania* spp. genes. In case of failure, keywords of the current annotation of SBP genes of *T. cruzi* were used as gene text during search. Ergosta-7,22-dien-3β-ol was a unique case; keyword “ergosta” was used to search Kyoto Encyclopedia of Genes and Genomes (KEGG) website (http://www.genome.jp/kegg/, latest access date 01/30/16) to find KEGG reactions and enzymes. Afterwards, keyword “hydroxysteroid” was used to identify genes in TriTryp database. Three genes were identified in *L. infantum*, one of which was a conserved hypothetic protein. The protein sequence was used to BLAST and a putative hydroxy steroid dehydrogenase/isomerase was identified. Accession numbers for individual genes were identified by BLAST search.

## Results

### Sterol identification

**Fig. 1:**
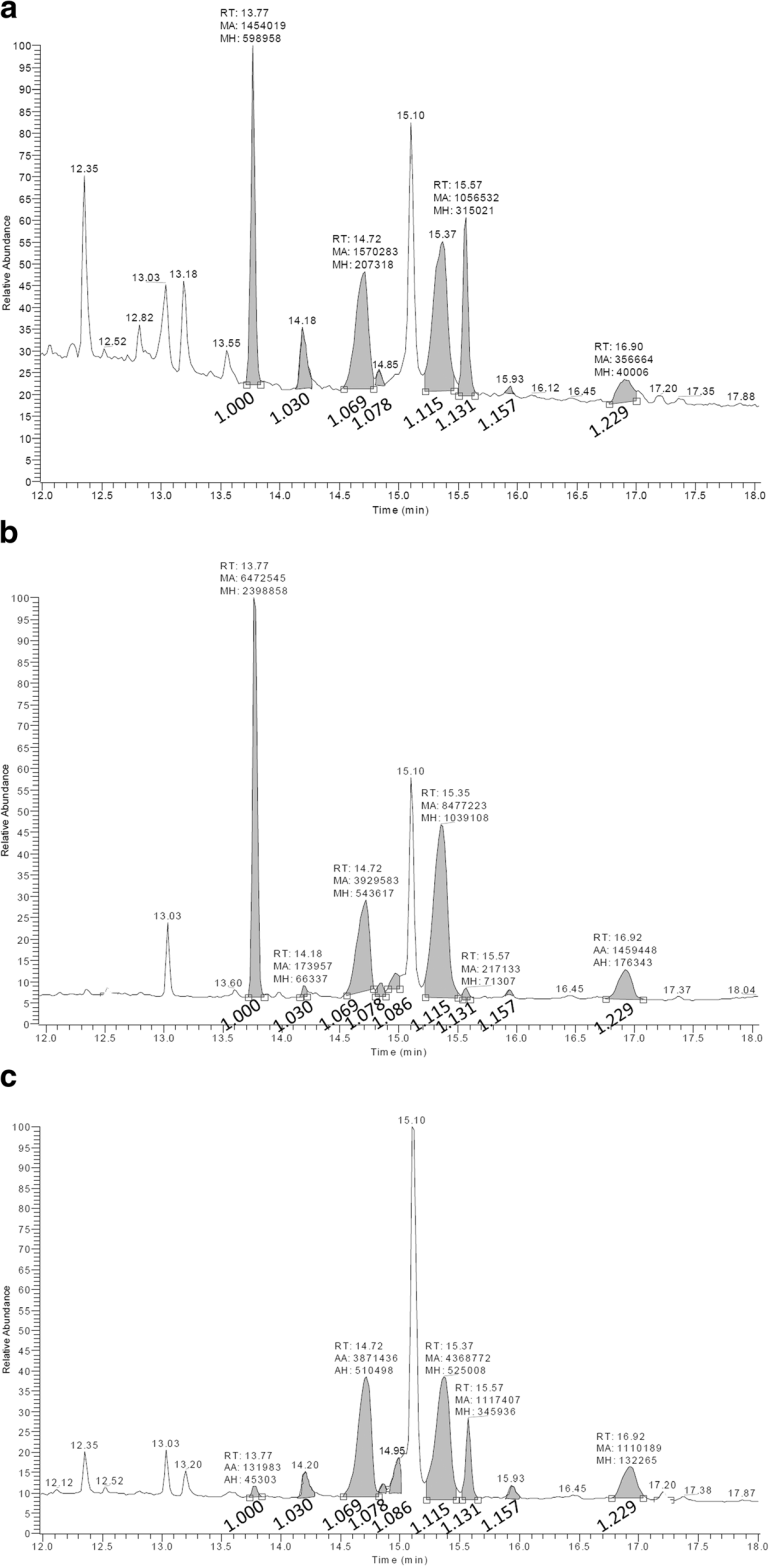
Representative gas chromatography spectra (GC) of total sterols of *Leishmania infantum* virulent and avirulent strains cultured in complete HOMEM or serum-free medium (SFM). Shaded peaks were identified as sterols with relative retention time to cholesterol as marked. 1.000-Cholesterol; 1.030-Zymosterol; 1.069-Ergosterol – I; 1.078-Ergosta-7,22-dien-3β-ol – I; 1.086-Ergostatetraenol; 1.115-Ergosterol – II; 1.131-Ergosta-7,22-dien-3β-ol – II; 1.157-Lanosterol; and 1.229-Stigmasta-7,24(28)-dien-3β-ol. **a** Stationary promastigotes of virulent strain cultured in HOMEM. **b** Stationary promastigotes of avirulent L5 strain cultured in HOMEM. **c** Stationary promastigotes of virulent strain cultured in SFM. The GC spectra of metacyclic promastigotes had been previously published [8]

We previously identified five sterols species from infectious *L. infantum* metacyclic promastigotes cultured in complete HOMEM in vitro [8]. The current investigations included logarithmic, stationary and metacyclic stage promastigotes in virulent (recent isolate) and avirulent L5 strains maintained in either HOMEM or SFM. All five of the previously identified sterols (cholesterol, two isomers of ergosterol with distinct GC retention time, ergosta-7,22-dien-3β-ol, and stigmasta-7,24(28)-dien-3β-ol) were identified in addition to four new sterols (ergostatetraenol, an additional isomer of ergosta-7,22-dien-3β-ol, zymosterol and lanosterol; Table 1; Fig. 1).

**Table 1:** Sterols identified by gas chromatograph-mass spectrometry (GC-MS) in promastigotes of *Leishmania infantum* derived from in vitro culture

Sterol	Identification^a^	RR_t_^b^
Cholesterol	I	1.000
Zymosterol	New	1.030
Ergosterol - I	II	1.069
Ergosta-7,22-dien-3β-ol - I	New	1.078
Ergostatetraenol	New	1.086
Ergosterol - II	III	1.115
Ergosta-7,22-dien-3β-ol - II	VI	1.131
Lanosterol	New	1.157
Stigmasta-7,24(28)-dien-3β-ol	V	1.229

^a^I-V were identified previously [8]. New indicates sterols identified in the current study

^b^Relative retention time (RRt) to cholesterol on GC, which is the ratio of retention time of each sterol to that of cholesterol

### Dynamic changes in sterols during promastigote metacyclogenesis

Discovery of sterols that are differentially represented in metacyclic versus less virulent promastigotes may provide clues to the differentiation and pathogenicity of the metacyclic promastigotes. Therefore sterols were isolated from a recent isolate of virulent *L. infantum* promastigotes cultured in HOMEM with 10 % FCS in vitro, in either logarithmic (day 3 to 4 of culture) or metacyclic stage. Metacyclic promastigotes were isolated from day 8 stationary-phase promastigotes.

**Fig. 2:**
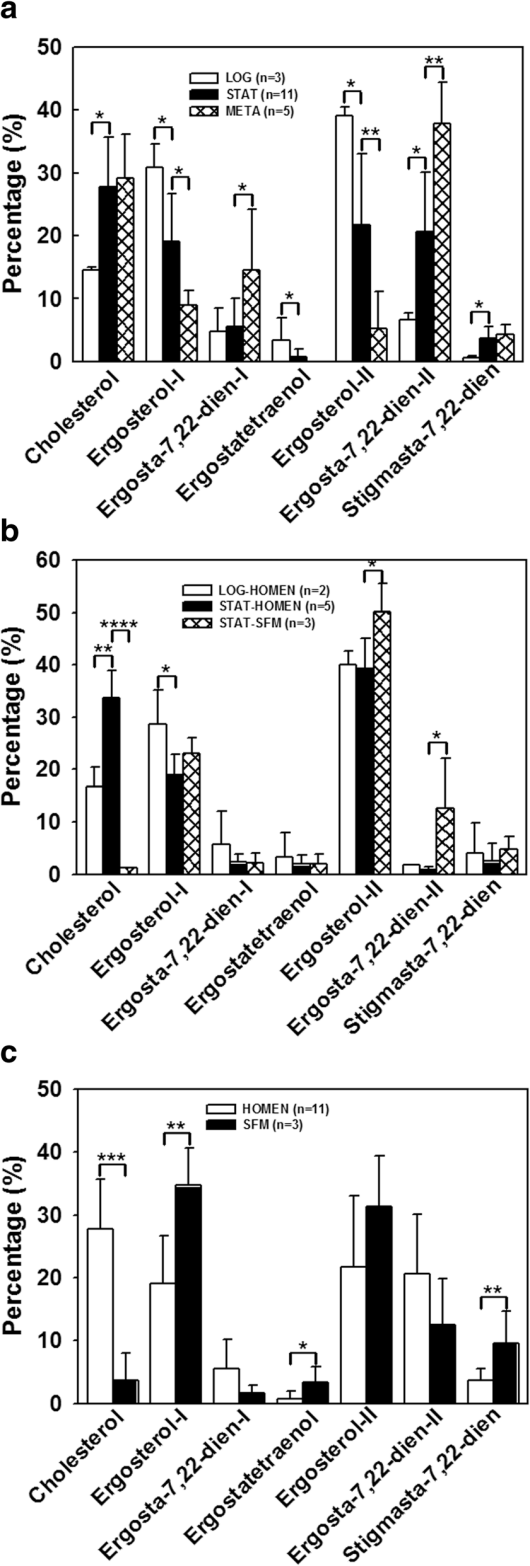
Sterol amount in percentage (%) among logarithmic, stationary and metacyclic promastigotes of *L. infantum* virulent or avirulent L5 strain grown in complete HOMEM or serum-free medium (SFM). LOG: logarithmic phase promastigotes; STAT: stationary phase promastigotes; META: metacyclic promastigotes. For each developmental stage, all sterols were added up to 100 %. **a** Virulent strain grown in HOMEM. **b** Avirulent L5 strain grown in HOMEM or SFM. **c** Stationary phase promastigotes of virulent strain cultured in HOMEM or SFM. *P < 0.05; **P < 0.01; ***P < 0.001; ****P < 0.0001

The dominant sterols in logarithmic-phase promastigotes were ergosterols. Ergosterol-I and ergosterol-II, two isomers with distinct retention times on the GC column, accounted for 30.92 % and 39.13 % of total sterols, respectively (Fig. 2a). The third most abundant sterol was cholesterol, accounting for 14.60 % of total sterols. In contrast, in stationary-phase promastigotes the two ergosterol isomers accounted for only 19.09 % and 21.74 %, respectively of total sterols, whereas cholesterol was the most abundant and constituted 27.77 % of total sterols (Fig. 2a). Metacyclic promastigotes were different from both; the two ergosterols accounted for only 8.94 % and 5.18 %, respectively, of total metacyclic promastigotes sterols. The two most abundant sterols in metacyclics were ergosta-7,22-dien--3β-ol-II and cholesterol, accounting for 37.90 % and 29.11 % of total sterols, respectively. The differences in sterols among the various promastigote stages were statistically significant (Fig. 2a), suggesting a stage-specific expression of various sterols in virulent *L. infantum* promastigotes.

We also analyzed sterols of a *L. infantum* avirulent L5 strain, which was derived by continuous culturing in vitro of a wild-type virulent strain for over 5 years as previously described [14]. As previously shown L5 appeared to undergo no metacyclogenesis, since almost no metacyclic promastigotes were recovered from stationary-phase L5 promastigotes (< 0.1 % recovery rate, compared to 13.4 % in the wild-type strain) [3]. In this strain, the most abundant sterols in both log and stationary phase promastigotes were ergosterol-II, accounting for 39.93 % and 39.28 % of total sterols, respectively (Fig. 2b). In log phase promastigotes the next two most abundant sterols were ergosterol-I (28.68 %) and cholesterol (16.68 %). In stationary cells, they were cholesterol (33.69 %) and ergosterol-I (18.99 %) (Fig. 2b). The difference between these two stages were statistically significant (Fig. 2b), indicating there is stage-specific variation in sterols in avirulent *L. infantum*, even in the absence of the capacity to undergo metacyclogenesis.

### Cholesterol in the culture medium affected sterol composition in *L. infantum* promastigotes

Trypanosomatid protozoa do not have the enzymes to synthesize cholesterol, and it has been shown that the bloodstream form of *T. brucei* can take up cholesterol from its environment [4]. As such, we hypothesized that the *Leishmania* culture medium might be an important determinant of the protozoan sterol composition. Promastigotes were cultured in parallel in either HOMEM supplemented with 10 % FCS, thus bathing the parasites in an exogenous source of cholesterol, or in a defined SFM that was similar to HOMEM but lacked serum (and thus lacked cholesterol). Sterols from stationary growth-phase promastigotes incubated in these media were compared. In virulent strain *L. infantum,* cholesterol accounted for 27.77 % and 3.69 % of total sterols in parasites grown in the serum-containing or serum-free media, respectively (*P* < 0.001, Fig. 2c). The proportions were 33.69 % and 1.14 % in avirulent L5 strain *L. infantum*, respectively (*P* < 0.0001, Fig. 2b). These data verified that cholesterol was reduced to minimal amounts in promastigotes incubated in a cholesterol-low environment, while other compounds were not as dramatically altered. Whether these were compensatory changes or merely changes in proportions required us to study the absolute cholesterol amounts, below.

### Sterols in virulent versus avirulent strain of *L. infantum*

Qualitative and quantitative differences in sterol composition between virulent and avirulent *Leishmania* spp. have not been reported, nor has it been determined whether virulence affects the ability of promastigotes to adsorb cholesterol from their environment. We therefore compared the relative amounts of each sterol component in log or stationary phase virulent or avirulent strains of *L. infantum,* cultured in either cholesterol-containing HOMEM or in cholesterol-free SFM. The results are summarized in Table 2. Several differences were observed. First, both virulent and avirulent *L. infantum* were able to adsorb cholesterol from the environment. Second, the amount of ergosterol-I in virulent *L. infantum* grown in SFM was proportionally greater than the avirulent L5 strain, with the difference made up by a relative decrease in ergosterol-II. Ergosterol-II was also decreased in virulent stationary parasites incubated in HOMEM, but in this case it was compensated by a significant increase in ergosta-7,22-dien-3β-ol compared to the avirulent L5 strain in either log or stationary growth phase (Table 2). These data demonstrate a difference in sterol synthesis between virulent and avirulent strains that extends beyond the ability to adsorb cholesterol from the external environment.

**Table 2:** Relative sterol content of a recently isolated virulent (vir) strain compared to an avirulent (L5) strain of *Leishmania infantum*

Parasite-growth phase	L5-LOG (*n =* 2)	Vir-LOG (*n =* 3)	L5-STAT (*n =* 5)	Vir-STAT (*n =* 11)	L5-STAT (*n =* 3)	Vir-STAT (*n =* 3)
Growth Medium	HOMEM				SFM	
Sterol:						
Cholesterol	16.68 (3.70)	14.60 (0.38)	33.69 (5.25)	27.77 (7.94)	1.14 (0.13)	3.69 (4.28)
Zymosterol	0.00 (0.00)	0.00 (0.00)	0.27 (0.38)	0.71 (2.17)	0.72 (0.63)	2.52 (2.25)
Ergosterol -I	28.68 (6.48)	30.92 (3.73)	18.99 (3.85)	19.09 (7.59)	22.96 (3.09)	34.70* (5.98)
Ergosta-7,22-dien-3β-ol - I	5.62 (6.48)	4.76 (3.74)	2.29 (1.58)	5.50 (4.64)	2.16 (1.94)	1.64 (1.33)
Ergostatetraenol	3.27 (4.62)	3.38 (3.57)	1.92 (1.72)	0.72 (1.25)	1.97 (1.80)	3.39 (2.39)
Ergosterol -II	39.93 (2.62)	39.13 (1.37)	39.28 (5.81)	21.74** (11.33)	50.17 (5.33)	31.33* (8.09)
Ergosta-7,22-dien-3β-ol- II	1.71 (0.04)	6.58* (1.20)	0.84 (0.66)	20.66*** (9.43)	12.69 (9.50)	12.56 (7.28)
Lanosterol	0.00 (0.00)	0.00 (0.00)	0.16 (0.36)	0.15 (0.35)	1.14 (1.07)	0.58 (1.00)
Stigmasta-7,24(28)-dien-3β-ol	4.10 (5.80)	0.63 (0.32)	2.50 (3.44)	3.65 (1.88)	4.77 (2.50)	9.60 (5.17)

Data show the average (standard deviation) percentages of each sterol relative to the total amount of sterols in each cell type, set at 100 %. Sterols were identified and quantified by gas chromatograph-mass spectrometry. *L. infantum* virulent (vir) or avirulent L5 promastigotes were cultured in either HOMEM or serum free medium (SFM) and harvested for sterol analyses in logarithmic (LOG) or stationary (STAT) growth phases

*P < 0.05; **P < 0.01; ***P < 0.001

### Dynamic changes in absolute amounts of sterols during promastigote metacyclogenesis

The above proportional differences in sterol content cannot reveal whether some parasite forms are relatively depleted of membrane sterols. We therefore measured absolute sterol amounts either directly or indirectly as described above. Surprisingly, stationary-phase promastigotes cultured in HOMEM had roughly two times the total sterol content compared to purified metacyclic promastigotes. The most dramatic differences were in cholesterol, and ergosterol-I and -II (Table 3). Depletion of sterols in the metacyclic promastigotes by MβCD treatment validated the measurements of sterols in other forms.

**Table 3:** Absolute amounts of sterols of stationary and metacyclic promastigotes of a virulent strain of *Leishmania infantum,* incubated in the absence or presence of MβCD^a^

Sterol	Stationary (*n =* 6)	Metacyclic (*n =* 6)	MβCD-metacyclic (*n =* 6)
Cholesterol	448.3 (249.2)	248.9 (120.8)	89.0 (40.6)*
Ergosterol -I	331.0 (290.5)	68.4 (13.1)	40.3 (22.3)*
Zymosterol	44.2 (108.2)	0.0 (0.0)	0.0 (0.0)
Ergosta-7,22-dien-3β-ol - I	81.8 (40.6)	100.6 (45.8)	49.3 (17.2)*
Ergostatetraenol	0.0 (0.0)	0.0 (0.0)	0.0 (0.0)
Ergosterol - II	403.6 (437.7)	35.7 (33.4)	11.5 (9.3)
Ergosta-7,22-dien-3β-ol - II	387.8 (282.1)	318.9 (137.8)	81.8 (37.3)**
Lanosterol	6.4 (15.7)	0.0 (0.0)	0.0 (0.0)
Stigmasta-7,24(28)-dien-3β-ol	62.8 (63.6)	37.8 (19.4)	17.5 (8.6)*
Total	1766.0 (1307.8)	810.2 (239.2)	289.5 (120.7)**

^a^Numbers indicate the mean (SD) absolute concentrations of sterols (ng/10^7^ cells). Metacyclic promastigotes were isolated from stationary phase promastigotes as previously described [3]. An aliquot of the metacyclic cells were treated with 25 mM MβCD

*P < 0.05; **P < 0.01 between metacyclic and MβCD-treated metacyclic promastigotes

### Absolute sterol content in avirulent or virulent parasites in different culture media

Absolute quantification revealed that total sterols were higher in either virulent or avirulent *L. infantum* strains when cultured in a cholesterol-containing medium (HOMEM) than in medium without cholesterol (SFM). Furthermore, virulent parasites contained more total sterols than the avirulent L5 strain grown in either medium. In virulent strain *L. infantum* promastigotes, the amount of cholesterol in stationary phase promastigotes was about 25 times higher when cultured in HOMEM (448.3 ng) than in SFM (18.0 ng). Nevertheless the cells in SFM synthesized more ergosterol-I and -II, i.e., 1001.8 ng in SFM compared to 734.6 ng in the HOMEM (Additional file 1: Table S1). In avirulent L5 strain *L. infantum*, the cholesterol amount was also many-fold higher when promastigotes were cultured in HOMEM than in SFM. Interestingly and in contrast to the virulent strain, L5 promastigotes in SFM appeared not to have synthesized more ergosterols despite cholesterol depletion (Additional file 1: Table S1).

### *Leishmania* spp. sterol biosynthetic pathway (SBP)

**Fig. 3:**
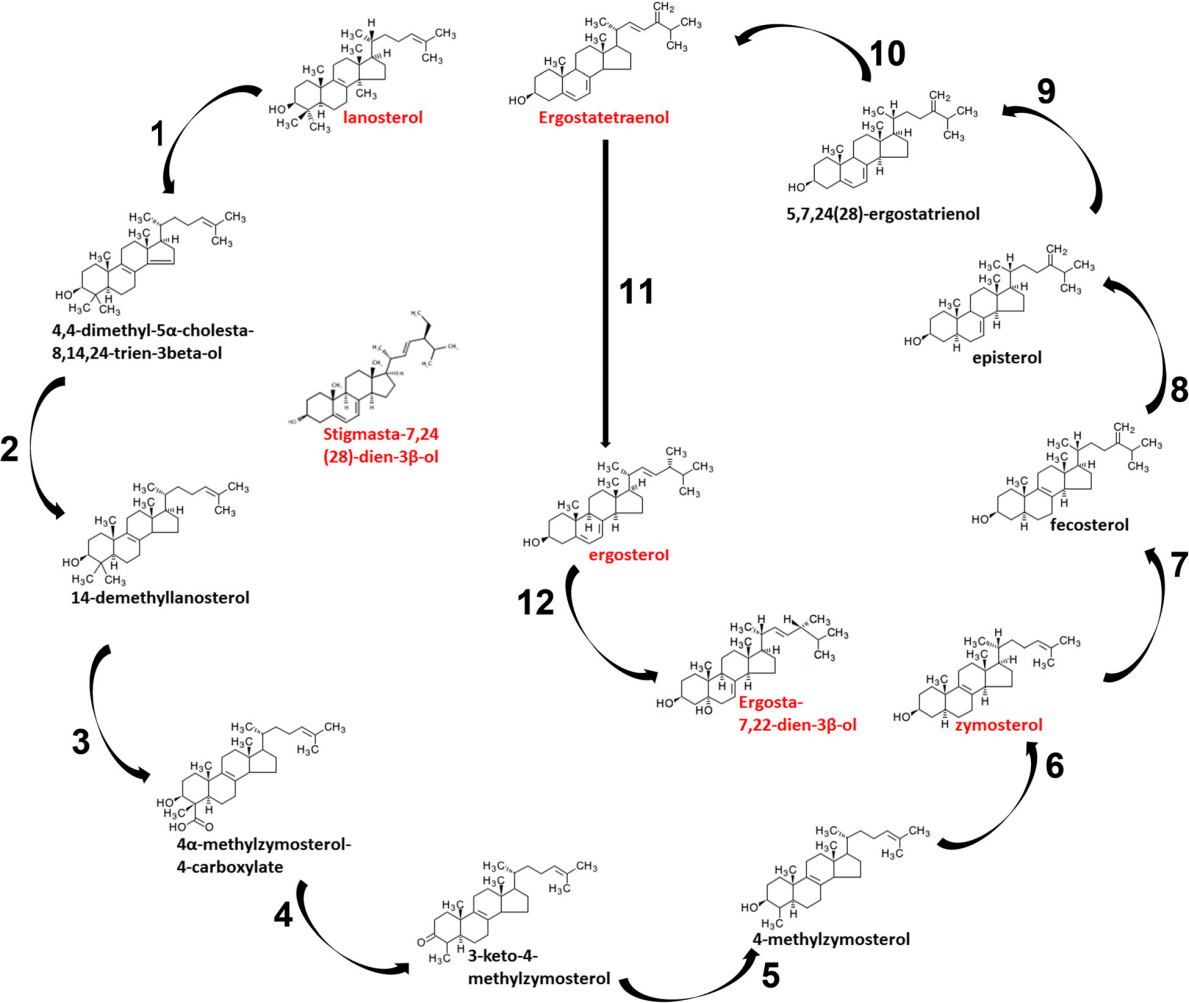
Sterol biosynthetic pathway of *Leishmania* spp. Sterols identified in the current study are labeled in red. Enzymes involved in each step of reaction are: 1, Lanosterol 14-α-demethylase; 2, C-14 sterol reductase; 3, C-5 sterol desaturase; 4, NAD(p)-dependent steroid dehydrogenase-like protein; 5 & 6, unknown; 7, Sterol 24-c-methyltransferase; 8, C-8 sterol isomerase-like protein; 9, Lathosterol oxidase-like protein; 10, Cytochrome p450-like protein; 11, Sterol C-24 reductase; 12, Hydroxysteroid dehydrogenase/isomerase

Based on the *Trypanosoma cruzi* genome, homologous SBP genes of *L. infantum* were identified for the multiple reactions from farnesyl diphosphate to ergosterol. Furthermore, an enzyme was identified for the reaction between ergosterol and ergosta-7,22-dien-3β-ol. Consequently all identified sterols reported in the current study were fitted in an SBP as shown in Fig. 3. One exception was stigmasta-7,24(28)-dien-3β-ol. No entry was found in either the TriTryp or the KEGG databases using keyword “stigmasta”. Corresponding genes and accession numbers of other *Leishmania* spp., including but are not limited to *L. donovani*, *L. major*, *L. braziliensis*, *L. mexicana* and *L. panamensis,* were also identified (Additional file 2: Table S2).

## Discussion

The genomes of the trypanosomatid protozoans, including *Trypanosoma* spp. and *Leishmania* spp., have genes encoding enzymes that catalyze the biosynthesis of ergosterol but not cholesterol [18–21]. This characteristic is more similar to fungi than other eukaryotes including mammals [22]. Consequently, amphotericin B, which selectively binds to ergosterol leading to disruption of the osmotic integrity of the membrane in target cells [12], is a powerful agent used for treatment of leishmaniasis and fungal infections of medical and veterinary importance. Amphotericin B is widely used as a first-line anti-leishmanial agent in patients from India where antimony-resistant strains of *Leishmania* spp. are the rule, or in the case of HIV - *Leishmania* spp. co-infections [10, 23].

Goad and colleagues (1984) identified major sterols of promastigotes of *L. tropica*, *L. donovani*, *L. mexicana* and *L. major* by GC-MS[5], and Xu and colleagues recently quantified sterols of *L. major* [13]. In keeping with the efficacy of treatment modalities, transient treatment with miltefosine, the first orally active anti-leishmanial drug effective in VL, significantly affects sterol content of *L. donovani* promastigotes. In one study, cholesterol and cholesta-5,7,24-trien-3β-ol were 2× and 32× higher, respectively, whereas ergosta-7,24(28)-dien-3β-ol and ergosta-5,7,24(28)-trien-3β-ol were 15× and 5 × lower, respectively, in miltefosine treated than untreated promastigotes [24]. Similarly, other anti-leishmanial agents such as chalcone and azasterols changed sterol profiles in *L. amazonensis* [25, 26]. Drug resistance is associated with significantly altered sterol profiles (e.g. atovaquone-resistant *L. infantum* [16], miltefosine-resistant *L. donovani* [27]).

Despite their inability to synthesize the compound, Trypanosomatids have ample cholesterol when measured [28], leading to questions about the relative importance of different sterols in pathogenesis. Herein we describe qualitative and quantitative analyses of sterol content in *L. infantum* promastigotes, the causative agent of VL. Our data suggest there is substantial adsorption of cholesterol from the parasite’s external environment, complicating interpretation of sterol content. To the best of our knowledge, this is the first report of the absolute amounts of sterols in parasites that differ in virulence and in developmental stage. We also make the unique observation that the sterol content is dramatically altered in different environmental media.

Sterols are identified by their relative retention time (RRt) observing their mass spectrum peak on gas chromatography. Goad and colleagues (1984) measured sterols in logarithmic-phase promastigotes of several *Leishmania* spp., reporting only trace amounts of cholesterol in cells cultured in lipid-free medium [5]. Ergosta-5,7,24(28)-trien-3β-ol was the dominant sterol among various species of *Leishmania* spp., ranging from about 60 % in *L. major*, 70 % in *L. tropica*, *L. mexicana* and *L. donovani* and up to 90 % in *L. amazonensis* and *L. pifanoi* [5]. Consistent with that report, we observed that cholesterol comprised less than 4 % of total sterols in promastigotes cultured in cholesterol-free SFM, with a much higher representation of ergosterol. This was in contrast to much higher percentages of cholesterol in promastigotes cultured in HOMEM containing FCS as a cholesterol source, confirming the ability of parasites in either growth stage to adsorb cholesterol from their external medium. In addition we detected eight sterols other than cholesterol, all presumptively precursors or derivatives of ergosterol (Fig. 3) (discussed below). These observations are consistent with the conclusion that ergosterol is the major sterol synthesized by *Leishmania* spp. with or without cholesterol in the external environment.

Less abundant sterols included ergosta-7,22-dien-3β-ol (20.7 %), stigmasta-7-24(28)-dien-3β-ol, ergostatetraenol, zymosterol and lanosterol, all less than 4 %. The last three had been found to be precursors of ergosterol in *Leishmania* spp., *T. brucei*, *T. cruzi* and the yeast *Saccharomyces cerevisiae* [4, 17, 29, 30]. Ergosta-7,22-dien-3β-ol has been previously detected in *L. donovani* and *L. amazonensis* [24, 26, 27] as well as in *S. cerevisiae* yeast and *Ganoderma pfeifferi* fungus [31, 32]. How it fits to the SBP has not been established. We identified an enzyme, hydroxysteroid dehydrogenase/isomerase (Accession number: XP_001464825.1) in the *Leishmania* genome, which could potentially catalyze a reaction linking ergosta-7,22-dien-3β-ol to ergosterol (Fig. 3). Stigmasta-7–24(28)-dien-3β-ol has been found in the fungus *Gymnosporangium juniper* [33]; ergostatetraenol in *Candida albicans* and *S. cerevisiae* [34, 35]; ianosterol in the woody plant pathogen *Eutypa lata* [36]; and zymosterol in *S. cerevisiae* [35].

Further discussion of stigmasta-7–24(28)-dien-3β-ol is warranted. This sterol has not been found in *T. brucei* [29] although it is often found in fungi and plants. This observation might have several explanations. First, the validity of stigmasta-7–24(28)-dien-3β-ol identification must be considered. The GC-MS peaks favorably argues for its identification, which is shown in the Additional file 3: Fig. S1. Secondly, this sterol might be taken up by *Leishmania* spp. promastigote from FCS, where it could be present in trace amounts. Our data do not support such a scenario. As showed in Table 2 promastigotes cultured in SFM had higher levels of this sterol compared to the cells derived from HOMEM. Thirdly, biosynthetic enzymes for this sterol might be unique to *Leishmania* spp. Stigmastanol is formed from either β-sitosterol by reduction, or through hydrogenation of stigmasterol.

Stigmasterol and sitosterol are phytosterols derived from mevalonate biosynthetic pathways. Isoprenoid synthesis is controlled by 3-hydroxy-3-methylglutaryl-CoA reductase (HMG-CoA reductase). The first committed step toward phytosterol biosynthesis is SMT1 (C-24 sterol methyl transferase), and the subsequent action of SMT2, which catalyzes a methyl transfer to methylene lophenol, directs the product toward sitosterol and stigmasterol biosynthesis. The annotated genomes of both *L. infantum* and *T. brucei* include genes predicted to encode a HMG-CoA reductase (LinJ.30.3230 and Tb927.6.4540). Two genes for a sterol 24-C methyl transferase are found in the *L. infantum* JPCM5 genome: LinJ.36.2510 and LinJ.36.2520, as is a sterol C-24 reductase (LinJ.33.0730) and several other (C-8, C-14) sterol reductases. The *T. brucei* genome also encodes two sterol 24-C-methyl transferases (TB11.v5.0496, Tb927.10.6950) and only one annotated (C-14) sterol reductase. Whether the enzymes of *L. infantum* participate in stigmastanol synthesis, and whether the additional sterol reductase genes present in *L. infantum* over *T. brucei* are influential in the differential recognition of stigmastanol only in *L. infantum*, cannot be determined without further experimental evidence. ([37], trytrypdb.org).

This would not be the first discovery of a pathway in *Leishmania* spp. that is more closely related to fungal metabolic pathways than those of *Trypanosoma brucei*. Indeed, *T. brucei* take in iron primarily through a transferrin receptor, whereas the *Leishmania* species use a two-step surface ferric iron reductase (Fe^3+^ → Fe^2+^)/ferrous iron transferase [38–40].

Calculations of absolute amounts of each sterol allowed us to assess (1) whether the total sterol content is associated with parasite virulence, and (2) whether adsorbed cholesterol inhibits ergosterol synthesis. Relevant to the first question, the total amount of sterols in 1 × 10^7^ stationary-phase promastigotes was 1,766.0 ng, roughly double the amount in purified metacyclic promastigotes from the same culture (810.2 ng, Table 3). The difference was mainly accounted for by a reduction in ergosterol I and II, with a lowering of adsorbed cholesterol as a second contributory change. *L. infantum* stationary-phase promastigotes contain 41.4 % metacyclic cells [3], so the remaining non-metacyclic promastigotes must have accounted for the differences. This difference may be partially explained by a smaller body size of the metacyclic promastigotes observed in both flow cytometry [41, 42] and microscopy [3, 41, 43–46], although this cannot be the full explanation since there were proportional differences between sterols in the populations, and because most sterols are located at the cellular membrane [47, 48]. Nevertheless, the significant difference in proportions suggest differences in ergosterol synthesis may contribute. Metabolic changes during metacyclogenesis could in part be favored by the absence of purine, or by an acidic environment [49–51]. It is possible that other environmental conditions such as environmental cholesterol/sterol concentrations also favor metacyclogenesis.

Cholesterol and ergosterol are integral components of plasma membrane micro-domains called lipid rafts, also known as detergent-resistant membrane (DRMs) [52]. MβCD specifically chelates and depletes sterols from lipid rafts. In the current study we confirmed our earlier report that MβCD treatment of metacyclic promastigotes reduced the total amount of cellular sterols by two thirds. We previously reported that lipid-raft depleted metacyclic promastigotes exhibit increased sensitivity to complement medicated lysis in vitro and reduced infectivity for mice in vivo [8].

*Leishmania* spp. are inoculated by the sand fly into mammalian tissue in a pool of blood, a relatively cholesterol rich environment. Our observations raise the possibility that suspension in such an environment might alter the membrane sterol content of the inoculated parasite. Host cholesterol levels affect the outcomes of *Leishmania* spp. infections. It has been reported that high circulating lipid levels offer protection against *L. donovani* infection in a mouse model where mice were either provided a high-cholesterol (atherogenic) diet or underwent statin treatment [53]. Similarly, patients with active VL had a lower level of total cholesterols during acute disease than four months after VL resolution in the same patients, and lower cholesterol than healthy volunteer controls [54]. Whether transient exposure to this environment at the initial stage of infection affects the virulence of promastigotes is a matter of speculation. From the current work we can conclude that the sterol content of *Leishmania* promastigotes is dynamic and responds not only to environmental conditions, but also the developmental changes that occur during metacyclogenesis. These changes seem to be a result of both adsorption of cholesterol from the environment, and likely also ergosterol synthesis by the *Leishmania* spp. parasites. The interaction between sterol content and parasite virulence is a yet evolving story.

## Conclusions

Leishmaniasis causes 20,000 annual deaths and is a threat to 5 % of the populations of four continents worldwide. Its causative pathogens include more than 20 species of protozoan parasites in the genus of *Leishmania*, and there is an association between parasite species and clinical outcomes. The pathogenic mechanisms used by the parasite are incompletely understood, and a better understanding could speed both vaccine development and new drug discovery. We hypothesized that dynamic changes in sterol composition during parasite development contribute to development of virulent forms of the parasite that are the most infectious for mammalian hosts such as humans. Qualitative and quantitative measures of sterols using gas chromatograph-mass spectrometry enabled us to contrast sterols in infectious versus non-infectious parasite forms. Our data clearly showed a unique profile of sterols in the infectious parasites, and revealed that external environment is a major determinate of cellular sterol content. These discoveries warrant further investigation of roles of sterols in *Leishmania* spp. pathogenesis.

## Additional files

Additional file 1: Table S1. Effect of different culture media on absolute sterol content of stationary-phase promastigotes of a virulent strain compared to an avirulent strain of *Leishmania infantum* ^+^. (DOCX 13 kb) Additional file 2: Table S2. Homologous genes of *Leishmania* spp. sterol biosynthetic pathway (SBP). *Trypanosoma cruzi* SBP genes were used as gene ID during database search except those marked with an “*”, in which keyword search was used. Blank cells indicate data not available. (XLSX 17 kb) Additional file 3: Figure S1. Identification of stigmasta-7,24(28)-dien-3β-ol by GC-MS. A: GC-MS peaks of the compound at Relative retention time (RRt) of 1.229. B: Peaks of CAS #481-19-6, stigmasta-7,24(28)-dien-3β-ol. C: Comparison of the peaks in A and B and relative amount for each peak is shown. (PPTX 100 kb)

## Abbreviations

CL: cutaneous leishmaniasis
DRM: detergent resistant membranes
FCS: fetal calf serum
GC-MS: gas chromatograph-mass spectrometry
KEGG: Kyoto Encyclopedia of Genes and Genomes
MβCD: methyl-β-cyclodextrin
SFM: serum free medium
RRt: relative retention time
VL: visceral leishmaniasis.
