# Supplementary material for: Dynamics of sterol synthesis during development of Leishmania spp. parasites to their virulent form
Source: Parasit Vectors. 2016 Apr 12;9:200. doi: 10.1186/s13071-016-1470-0 (PMC4830053; doi:10.1186/s13071-016-1470-0)
Supplement: Additional file 1: Table S1. — Effect of different culture media on absolute sterol content of stationary-phase promastigotes of a virulent strain compared to an avirulent strain of Leishmania infantum +. (DOCX 13 kb) [file 13071_2016_1470_MOESM1_ESM.docx]

**Supplementary Table 1. Effect of different culture media on absolute sterol content of stationary-phase promastigotes of a virulent strain compared to an avirulent strain of *Leishmania infantum*^+^.**

|  | Virulent strain | | Avirulent L5 strain | |
| --- | --- | --- | --- | --- |
| Sterol | HOMEM (n=6) | SFM (n=2) | HOMEM (n=1) | SFM (n=2) |
| Cholesterol | 448.3 (249.2) | 18.0 | 352.5 | 3.9 |
| Ergosterol - I | 331.0 (290.5) | 467.5 (85.5) | 214.0 | 79.3 (27.0) |
| Zymosterol | 44.2 (108.2) | 34.4 (48.7) | 9.5 | 2.1 (3.0) |
| Ergosta-7,22-dien-3β-ol - I | 81.8 (40.6) | 27.7 (22.4) | 10.9 | 9.3 (5.8) |
| Ergostatetraenol | 0 | 61.6 (31.5) | 17.4 | 4.5 (6.4) |
| Ergosterol - II | 403.6 (437.7) | 534.3 (86.9) | 461.7 | 166.3 (41.7) |
| Ergosta-7,22-dien-3β-ol - II | 387.8 (282.1) | 219.4 (94.8) | 11.8 | 52.1 (28.9) |
| Lanosterol | 62.8 (63.6) | 13.8 (19.5) | 9.3 | 4.1 (5.7) |
| Stigmasta-7,24(28)-dien-3β-ol | 62.8 (63.6) | 106.4 (63.7) | 79.5 | 17.4 (14.8) |
| Total | 1766.0 (1307.8) | 1483.0 (155.6) | 1166.5 | 346.0 (54.2) |

^+^: Numbers indicate the mean (SD) absolute concentrations of sterols (ng/10^7^ cells) in stationary phase promastigotes of each strain cultured in different media. HOMEM has FCS, providing a source of exogenous cholesterol. SFM is cholesterol-free.
