# Supplementary figures and images for: Dynamics of sterol synthesis during development of Leishmania spp. parasites to their virulent form
Source: Parasit Vectors. 2016 Apr 12;9:200. doi: 10.1186/s13071-016-1470-0 (PMC4830053; doi:10.1186/s13071-016-1470-0)

## Slide 1
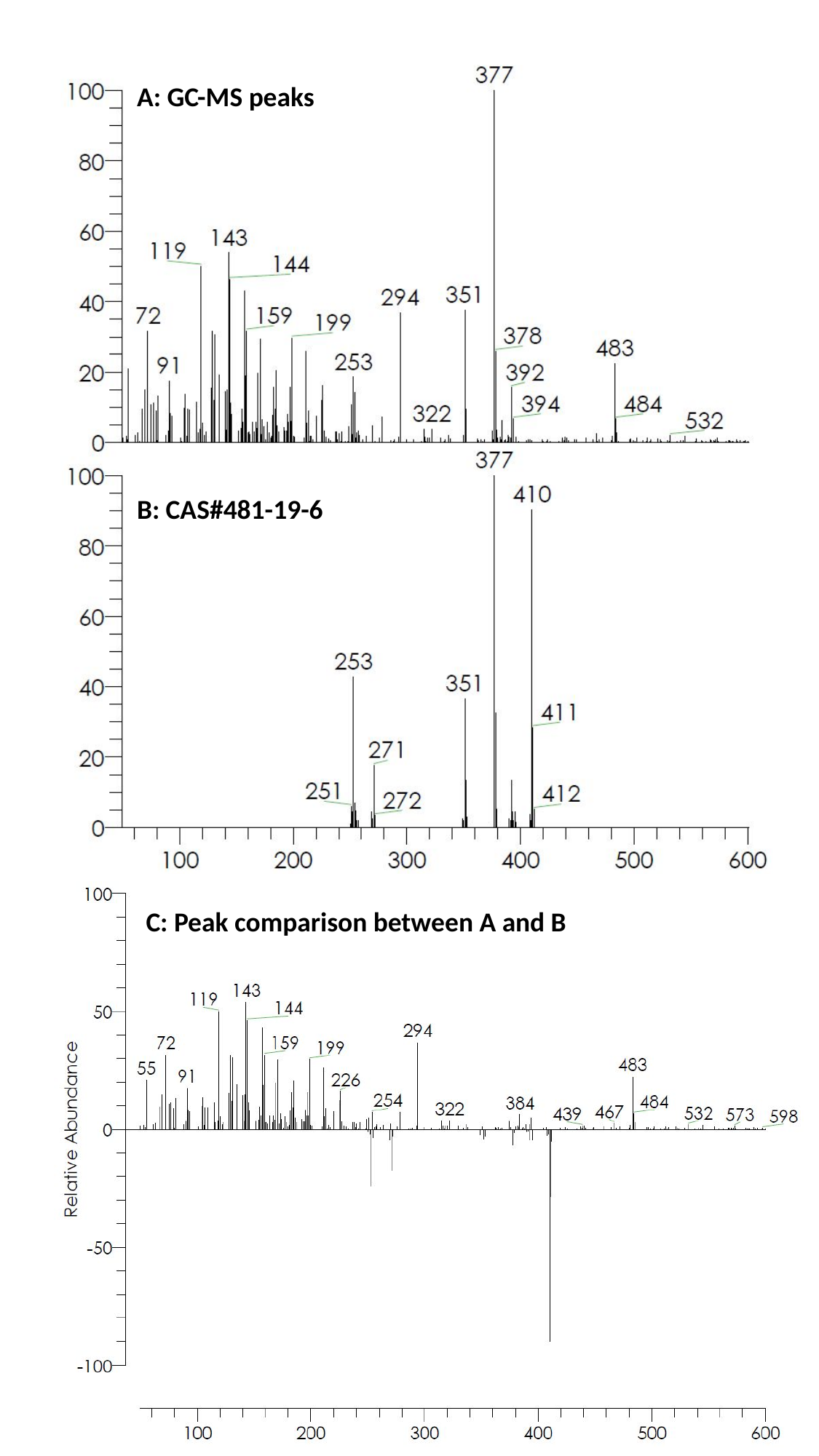

A: GC-MS peaks
B: CAS#481-19-6
C: Peak comparison between A and B

Supplement: Additional file 3: Figure S1. — Identification of stigmasta-7,24(28)-dien-3β-ol by GC-MS. A: GC-MS peaks of the compound at Relative retention time (RRt) of 1.229. B: Peaks of CAS #481-19-6, stigmasta-7,24(28)-dien-3β-ol. C: Comparison of the peaks in A and B and relative amount for each peak is shown. (PPTX 100 kb) [file 13071_2016_1470_MOESM3_ESM.pptx]
